# Clinically inapparent right heart dysfunction is associated with reduced myofilament force development in coronary artery disease

**DOI:** 10.1186/s12872-021-01926-6

**Published:** 2021-03-05

**Authors:** C. Bening, V. L. Sales, K. Alhussini, D. Radakovic, R. Cris Benitez, N. Madrahimov, D. Keller, R. Leyh

**Affiliations:** 1 Department of Thoracic and Cardiovascular Surgery, Zentrum Operative Medizin, University of Wuerzburg, Oberduerrbacherstr. 6, 97080 Wuerzburg, Germany; 2 Comprehensive Heart Failure Centre (CHFC) Würzburg, University of Wuerzburg, Wuerzburg, Germany

**Keywords:** Skinned fiber, Coronary artery bypass grafting, Right heart impairment, pCa

## Abstract

**Background:**

Right ventricular dysfunction after CABG is associated with poor peri- and postoperative outcomes. We aimed to identify clinical and experimental predictors for preoperative inapparent right ventricular dysfunction and therefore hypothesized that reduced myofilament force development as well as altered levels of biomarkers might predict inapparent right ventricular dysfunction.

**Methods:**

From 08/2016 to 02/2018, 218 patients scheduled for CABG were divided into two groups (TAPSE ≥ 20 mm, n = 178; TAPSE < 20 mm, n = 40). Baseline serum samples for biomarkers (Galectin, TGFß1, N Acyl-SDMA, Arginine, ADMA and Pentraxin-3), clinical laboratory and transthoracic echocardiographic parameters were evaluated. To examine the myocardial apparatus of the right ventricle intraoperative right auricular tissue was harvested for stepwise skinned fiber force measurements.

**Results:**

Patients with TAPSE < 20 mm had a higher incidence of DM (55 vs. 34%, p = 0.018), preoperative AFib (43 vs. 16%, p < 0.001), reduced GFR (67 ± 18 vs. 77 ± 24 ml/min/1.73 m^2^, p = 0.013), larger LA area (22 ± 6 vs. 20 ± 5 cm^2^, p = 0.005) and reduced LVEF (50 vs. 55%, p = 0.008). Furthermore, higher serum ADMA (0.70 ± 0.13 vs. 0.65 ± 0.15 µmol/l, p = 0.046) and higher serum Pentraxin-3 levels (3371 ± 1068 vs. 2681 ± 1353 pg/dl, p = 0.004) were observed in these patients. Skinned fiber force measurements showed significant lower values at almost every step of calcium concentration (pCa 4.52 to pCa 5.5, p < 0.01 and pCa 5.75–6.0, p < 0.05). Multivariable analysis revealed DM (OR 2.53, CI 1.12–5.73, Euro Score II (OR 1.34, CI 1.02–1.78), preoperative AF (OR 4.86, CI 2.06–11.47), GFR (OR 7.72, CI 1.87–31.96), albumin (OR 1.56, CI 0.52–2.60), Pentraxin-3 (OR 19.68, CI 14.13–25.24), depressed LVEF (OR 8.61, CI 6.37–10.86), lower force values: (pCa 5.4; OR 2.34, CI 0.40–4.29 and pCa 5.2; OR 2.00, CI 0.39–3.60) as predictors for clinical inapparent right heart dysfunction.

**Conclusions:**

These preliminary data showed that inapparent right heart dysfunction in CAD is already associated with reduced force development of the contractile apparatus.

## Background

Right ventricular dysfunction in patients undergoing coronary artery bypass surgery (CABG) is associated with poor perioperative and postoperative outcomes [1–3]. Identifying patients at risk for impaired right heart function could be helpful in risk-stratification of surgical candidates with symptomatic coronary artery disease (CAD) [3]. Due to the complex geometry of the right heart, its functional assessment is cumbersome [4, 5]. The ACC/AHA and ESC guidelines recommend TAPSE, for estimating RV global systolic function [5, 6]. However, earlier studies providing TAPSE cut-off at < 16 mm yielded low sensitivity in patients with reduced RV, highlighting the potential pitfall of this technique in demonstrating the involvement of intrinsic myocardial force capacity of right ventricular myofilaments [6]. Furthermore, underlying remodeling processes occurring in RV of preoperative CABG patients as well as those with reduced right heart function have not been well-defined utilizing prognostic biomarkers [7].

The relative paucity of translational studies on the relationship between TAPSE, intrinsic myocardial function and biomarkers at the level of the myocardial apparatus in symptomatic CAD patients prior to CABG, contribute to this uncertainty. We hypothesized that patients with impaired evident (TAPSE < 16 mm) and inapparent (TAPSE > 16 mm and < 20 mm) right heart dysfunction may have impaired induced myocardial force values and could be identified with altered levels of biomarkers underlying myocardial remodeling processes. For assessment of the myocardial contractility, we chose the skinned human fiber method, which is “a canonical functional probe of the intrinsic myocardial regulatory complex” and offers the possibility to examine the contractile properties of the myocardial apparatus (actin and myosin) directly by withdrawal of membrane-dependent processes [8–11].

This is important since membrane-dependent processes are likely to influence contractile kinetics by enzymatic modifications or membrane receptor mediated effects. This can be excluded in the skinned fiber mode, as direct observations of the myofilaments offer insights into the process of (impaired) contraction. Considering that atrial tissue has comparable contractile properties to ventricular tissue, results derived from skinned atrial tissue may also have relevance for the heart as a whole and is a well-established method in literature [12, 13].

The aim of the study was to evaluate if clinical inapparent or “low range normal” right ventricular function (TAPSE between 16 and 20 mm) is already associated with altered serum concentrations of selected biomarkers, altered right auricular force values (as signs of impaired function of the contractile apparatus) and clinical parameters of the patients.

## Methods

Two-hundred eighteen patients undergoing elective on pump CABG between August 2016 and February 2018 were enrolled in this study. All patients were informed and gave their written consent to participate. The study was approved by the University Hospital ethics committee (IRB approval: 143/17-sc 6.10.2017). Patients with valve pathologies, reoperations, emergency indications or pulmonary hypertension of any origin were excluded.

All patients received a routine preoperative echocardiographic examination (Philips Epiq 7, Philips Health System, Hamburg, Germany). Measurements and analysis were performed by trained echocardiography technician, blinded to patients. We measured left and right atrial end-systolic areas, systolic pulmonary artery pressure (sPAP), TAPSE, left ventricular ejection fraction (LVEF), according to echocardiographic guidelines for assessment of right heart function [10]. We divided the patients in two groups: group 1 with TAPSE values ≥ 20 and group 2 with TAPSE value < 20 mm and compared experimental force values from the right atrial auricle as well as all baseline clinical characteristics among both groups. All data including clinical findings and preoperative medication were recorded pseudonymously in a departmental database. Prior to induction of anesthesia, preoperative routine blood samples were drawn from all patients and immediately sent to the laboratory to be stored at − 80 °C.

Preoperative clinical chemistry included creatinine, albumin, glomerular filtration rate (GFR) and N-terminal pro brain natriuretric peptid (NT-proBNP). We performed enzyme-linked immunosorbant assay (ELISA) for the following markers of fibrosis and inflammation, taken from serum blood samples of the patients. All ELISAs were conducted with the ELISA reader Tecan Infinity Pro (Tecan Group Ltd., Switzerland): Galectin, Transforming Growth Factor ß (TGFß), N Acyl-Symmetric Dimethylarginine (N-Acyl-SDMA), Arginine, Asymmetric Dimethylarginine (ADMA) (Quantikine Elisa, DGD150, R&D Systems, Bio-Techne, Wiesbaden, Germany) and Pentraxin (Pentraxin 3 Human ELISA, RD191477200R, BioVendor, Czech Repuplic).

### Experimental set-up

We measured the calcium-induced force of right atrial skinned human fibers as previously described [8–10]. We resected the RAA for venous cannulation of extracorporal circulation. This RAA tissue was used for experimental calcium-induced force measurements. Briefly, the intraoperatively resected tissue was transported in ice-cold oxygenated cardioplegic solution, containing BDM (Sigma Aldrich Chemie GmbH, Steinheim, Germany). For the skinning procedure, the trabeculae were excised, immersed in Triton-X solution (Sigma Aldrich Chemie GmbH, Steinheim, Germany) and processed for sarcolemma removal.

The trabeculae were then cut small bundles for the experimental cycle. The experimental set-up has been previously described [8–10]. Briefly, the bundles were fixed between two forceps. A force transducer is connected to one of the forceps for recording any calcium-induced length changes. The bundles were then immersed increasing calcium concentrations. The calcium concentration is displayed as logarithmic calcium concentration (pCa), which is a negative decadic logarithm. We started with the lowest calcium concentration at pCa 7.0 and increasing at 6.5, 6.0, 5.75, 5.5, 5.4, 5.3, 5.2, 5.1, 5.0, 4.75, 4.52.

### Statistical analysis

Demographic variables, comorbidities, blood tests, ventricular function, operative data, 30-day, and mid-term outcomes in patients undergoing elective CABG were compared between patients with TAPSE ≤ 20 versus ≥ TAPSE. Continuous and categorical variables were compared using t-test and Fisher’s exact test, respectively. Multivariable linear and logistic regression models were constructed to identify factors associated with reduced right heart function (TAPSE ≤ 20 mm), postoperative atrial fibrillation (POAF) and length of stay. Candidate variables were selected a priori following a review of existing literature as well as the clinical and research experiences of the investigators [1, 8–10]. Moreover, we also performed the initial screening of variables using conventional multivariable linear and logistic binary regression models. By making the assumption of independence of echocardiographic TAPSE recordings within patients, we performed conservative entry/retention criteria and accordingly identified candidate covariates for analysis. Candidate variables considered for the multivariable analyses were those detected by univariate models as having a p < 0.05 or suggestive trend toward association (p = 0.05–0.20) predictive of abnormal TAPSE, postoperative atrial fibrillation (POAF) and length of stay; retention of variables in the multivariable model was set at p < 0.05. To discriminate independent risk factors, multivariable modeling was performed with methods of stepwise selection, with entry/stay criteria of 0.1/0.1 and exclusion of highly intercorrelated, redundant explanatory variables with r values set at > 0.70, with TAPSE groups and candidate variables all competing for entry into a final model. After excluding variables with significant intercorrelation, this analysis yielded 32 candidates for the multivariable model. Candidate variables include age, Euro Score II, diabetes mellitus (DM), preoperative AF, glycated haemoglobin (HbA1c), NTPro-BNP, GFR, albumin, ADMA, Pentraxin-3, right end-systolic area, sPAP,, left atrial diameter, LVEF, cardiopulmonary bypass (CPB) time, incision-suture time, ventilation time, prolonged ventilation, length of stay, postoperative atrial fibrillation (POAF) and pCa forced values. The final model included all independent risk factors for reduced right heart function, POAF and length of stay meeting these criteria. Multivariable linear regression was used to adjust for significant independent variables for reduced right heart function (TAPSE ≤ 20 mm) and length of stay and multivariable logistic regression was used to adjust for significant variables for POAF. All estimates are provided with 95% confidence intervals (CI). Probability p-values were considered as statistically significant when p < 0.05. All analyses were performed using SPSS 25 (IBM; Amonk, New York, USA).

### Clinical outcomes

Death notification was confirmed by medical record (evidence of life or death).

## Results

### Patient baseline and perioperative characteristics

218 patients who underwent elective CABG were evaluated. The age (mean ± SD) of the patient cohort was 68 ± 9 years and 187 (86%) were male. The clinical characteristics of patients are summarized in Table 1.

**Table 1:** Baseline Characteristics of 218 patients undergoing elective CABG

Characteristics	Total 218 (100%)	Group 1 n:178 (82%)	Group 2 n: 40 (18%)	P value
Age (y, mean ± SD	67.6 ± 9.4	67.1 ± 9.5	70.0 ± 8.5	**0.079**
Female (n,%)	31 (14.2)	24 (13.5)	7(17.5)	0.465
Height (cm, mean ± SD)	172.0 ± 7.4	172.0 ± 7.5	172.0 ± 6.8	0.855
Weight (kg, mean ± SD)	87.5 ± 15.8	87.3 ± 16.2	88.3 ± 14.3	0.721
EuroScore II (mean ± SD)	1.6 ± 1.2	1.4 ± 1.1	2.2 ± 1.6	** < 0.0001**
Diabetes Mellitus (n,%)	82 (37.6)	60 (33.7)	22 (55.0)	**0.018**
Hypertension (n,%)	188 (86.2)	155 (87.1)	33 (82.5)	0.450
Atrial fibrillation (n,%)	45 (20.7)	28 (15.8)	17 (42.5)	** < 0.0001**
HbA1c (%, mean ± SD)	6.2 ± 1.0	6.1 ± 1.0	6.5 ± 1.2	**0.020**
NTPro-BNP (pg/mL, mean ± SD)	964.8 ± 2879.0	853.2 ± 2988.4	1461.3 ± 2257.4	**0.129**
Creatinine (mg/dL, mean ± SD)	1.2 ± 0.7	1.1 ± 0.8	1.2 ± 0.4	0.726
GFR (ml/min/1.73 m^2^, mean ± SD)	74.8 ± 23.1	76.7 ± 23.8	66.7 ± 17.6	**0.013**
Albumin (g/dl, mean ± SD)	4.2 ± 0.5	4.3 ± 0.5	4.1 ± 0.4	**0.036**
Galectin (ng/dL, mean ± SD)	10.7 ± 5.4	10.6 ± 5.7	10.9 ± 4.3	0.842
TGFβ1 (pg/mL, mean ± SD)	29,570.6 ± 68,503.3	30,876.8 ± 75,129.3	23,757.9 ± 21,591.2	0.633
N Acyl-SDMA (µmol/l, mean ± SD)	0.69 ± 0.32	0.69 ± 0.34	0.73 ± 0.22	0.318
Arginin (µmol/l, mean ± SD)	92.9 ± 31.5	92.4 ± 31.6	94.8 ± 31.0	0.363
ADMA (µmol/l, mean ± SD)	0.66 ± 0.15	0.65 ± 0.15	0.70 ± 0.13	**0.046**
Pentraxin-3 (pg/dl, mean ± SD)	2807.4 ± 1329.2	2681.0 ± 1353.4	3370.5 ± 1068.4	**0.004**
RA area (cm^2^ mean ± SD)	15.9 ± 4.3	15.7 ± 3.8	16.5 ± 5.8	**0.089**
sPAP, mmHg	27.9 ± 8.5	27.4 ± 8.1	30.3 ± 10.1	**0.204**
LA area (cm^2^ mean ± SD)	20.4 ± 5.3	19.9 ± 5.0	22.4 ± 6.0	**0.005**
TAPSE	23.0 ± 4.0	24.3 ± 3.1	17.3 ± 1.7	** < 0.0001**
LVEF (%, mean ± SD)	53.7 ± 9.8	54.6 ± 9.3	49.7 ± 11.2	**0.008**

*FEV1* forced expiratory volume, *CRP* C-reactive protein, *HbA1c* glycated haemoglobin (HbA1c), *NTPro-BNP* N-terminal pro brain natriuretric peptid, *GFR* glomerular filtration rate, *TGF ß-1* Transforming Growth Factor ß, *IL-6* Interleukin-6, *N Acyl-SDMA* Symmetric Dimethylarginine, *ADMA* Asymmetric Dimethylarginine, *RA* right atrial, *sPAP* Systolic pulmonary artery pressure, *LA* left atrial, *LVEF* Left ventricular ejection fraction

Patients with reduced TAPSE (Group 2) presented with a significant higher Euro Score II (2.2 ± 1.6 vs. 1.4 ± 1.1, *p* < 0.001), higher incidence of DM (55% vs. 34%, *p* = 0.018), greater prevalence of AF (43% vs. 16%, *p* < 0.001), larger LA size (22 ± 6 cm^2^ vs. 20 ± 5 cm^2^, *p* = 0.005), decreased LVEF (LVEF 50% vs. 55%, *p* = 0.008), reduced preoperative glomerular filtration rates (GFR, MDRD: 66.7 ± 17.60 ml/min/1.73 m^2^ vs. 76.7 ± 23.8 ml/min/1.73 m^2^, *p* = 0.013), lower preoperative Albumin serum concentrations (4.1 ± 0.4 g/dl vs. 4.3 ± 0.5 g/dl, *p* = 0.036) and higher serum concentrations of Pentraxin-3 (3371 ± 1068 pg/dl vs. 2681 ± 1353 pg/dl, *p* = 0.004) and increased ADMA levels (0.70 ± 0.13 µmol/l vs. 0.65 ± 0.15 µmol/l, *p* = 0.046).

Operative and postoperative data are depicted in Table 2: Median bypass time was significant longer in patients in group 2 (114 (265) minutes vs. 107 (161) minutes, *p* = 0.009). Furthermore patients developed significant more often POAF (53 vs. 30%, *p* = 0.009) and prolonged LOS (mean 13 days vs. 11 days, *p* = 0.009). As of July 2019, the mean (± SD) follow-up was 14 ± 6 months in group 2 and 13 ± 6 months in group 1. Of the 218 patients, two died (0.9%) within 30 days of surgery while the overall follow-up rate for mid-term outcomes (> 30 days) presented in Table 2 among 30-day survivors was 98% (212/216). Late deaths (beyond 30 days post-surgery) have occurred in 5 patients during follow‐up (median, 13 months).

**Table 2:** Operative and early and midterm outcomes

Variables	Total n: 218 (100%)	Group 1 n: 178 (82%)	Group 2 n:40 (18%)	P value
*Intraoperative times*				
Cardiopulmonary bypass time (minutes, median [IQR])	108.0(265.0)	107.0(161)	114.0(265..0)	**0.009**
Cross Clamp time (minutes, median [IQR])	81.0(195.0)	82.0(195.0)	79.0(171.0)	0.540
Incision-suture time (minutes, median [IQR])	234.5(388.0)	235.5(292.0)	229.5(354.0)	**0.161**
*Early outcomes (≤ 30 d)*				
Ventilation time (hours,median [IQR])	9.3(398.7)	9.2(398.7)	10.8(195.1)	**0.127**
Prolonged Ventilation (≥ 12 h,n %)	68(31.2)	52(29.2)	16(40.0)	**0.191**
Length of stay (days, mean ± SD)	11.4 ± 5.1	10.9 ± 3.9	13.3 ± 8.3	**0.009**
Postoperativce Afib (n, %)	74(33.9)	53(29.8)	21(52.5)	**0.009**
Discharge and 30-d Mortality (n, %)	2(0.9)	2(1.1)	0(0.0)	1.000
*Mid-term outcomes (> 30d)*				
All-cause mortality (> 30-d, n, %)	5(2.3)	4(2.3)	1(2.5)	1.000

*IQR* Interquartile range

Table 3 demonstrates the experimental force measurements at increasing steps of calcium concentrations. Patients with reduced TAPSE had significant lower force values at almost every step of pCa except for the lowest pCa 7.0:0.021 ± 0.014 mN versus 0.023 ± 0.015 mN, p = 0.625) and the second lowest step of pCa 6.5 (0.035 ± 0.017 mN vs. 0.042 ± 0.022 mN, *p* = 0.069). At all other steps of calcium concentration were significant lower in the group with reduced TAPSE.

**Table 3:** Calcium-induced force values of right atrial myofilaments in 218 patients

Characteristics	Group 1 n: 178 (82%)	Group 2 n: 40 (18%)	P value
pCa 7.0	0.023 ± 0.015 mN	0.021 ± 0.014 mN	0.625
pCa 6.5	0.042 ± 0.022 mN	0.035 ± 0.017 mN	**0.069**
pCa 6.0	0.148 ± 0.088 mN	0.112 ± 0.052 mN	**0.011**
pCa 5.75	0.263 ± 0.162 mN	0.193 ± 0.111 mN	**0.010**
pCa 5.5	0.450 ± 0.242 mN	0.330 ± 0.183 mN	**0.004**
pCa 5.4	0.567 ± 0.272 mN	0.424 ± 0.222 mN	**0.002**
pCa 5.3	0.630 ± 0.296 mN	0.462 ± 0.246 mN	**0.001**
pCa 5.2	0.705 ± 0.326 mN	0.521 ± 0.264 mN	**0.001**
pCa 5.1	0.763 ± 0.343 mN	0.575 ± 0.282 mN	**0.001**
pCa 5.0	0.770 ± 0.358 mN	0.574 ± 0.929 mN	**0.001**
pCa 4.75	0.847 ± 0.375 mN	0.642 ± 0.304 mN	**0.001**
pCa 4.52	0.834 ± 0.384 mN	0.636 ± 0.315 mN	**0.003**

*pCa* logarithmic calcium-concentration

### Independent predictors of reduced TAPSE

Higher incidence of DM odds ratio (OR) 2.53; 95% CI, 1.12–5.73), higher EuroScore II (OR, 1.34; 95% CI, 1.02–1.78) greater prevalence of preoperative AF (OR ratio, 4.86; 95% CI, 2.06–11.47), decreased GFR (OR, 7.72; 95% CI, 2.06–11.47) and lower albumin level (OR, 1.56; 95% CI, 0.52–2.60), higher level of Pentraxin-3 (OR, 4.2719.68; 95% CI, 14.5813–1125.5124), poorer LVEF (OR, 8.61; 95% CI, 6.37–10.76) and lower calcium-induced force values: pCa 5.4 (OR, 2.34; 95% CI, 0.4–04.29 and pCa 5.2 (OR 2.00; 95% CI, 0.40–3.60 appear to influence the odds of reduced right heart function, represented by abnormal TAPSE (< 20 mm).

**Fig. 1:**
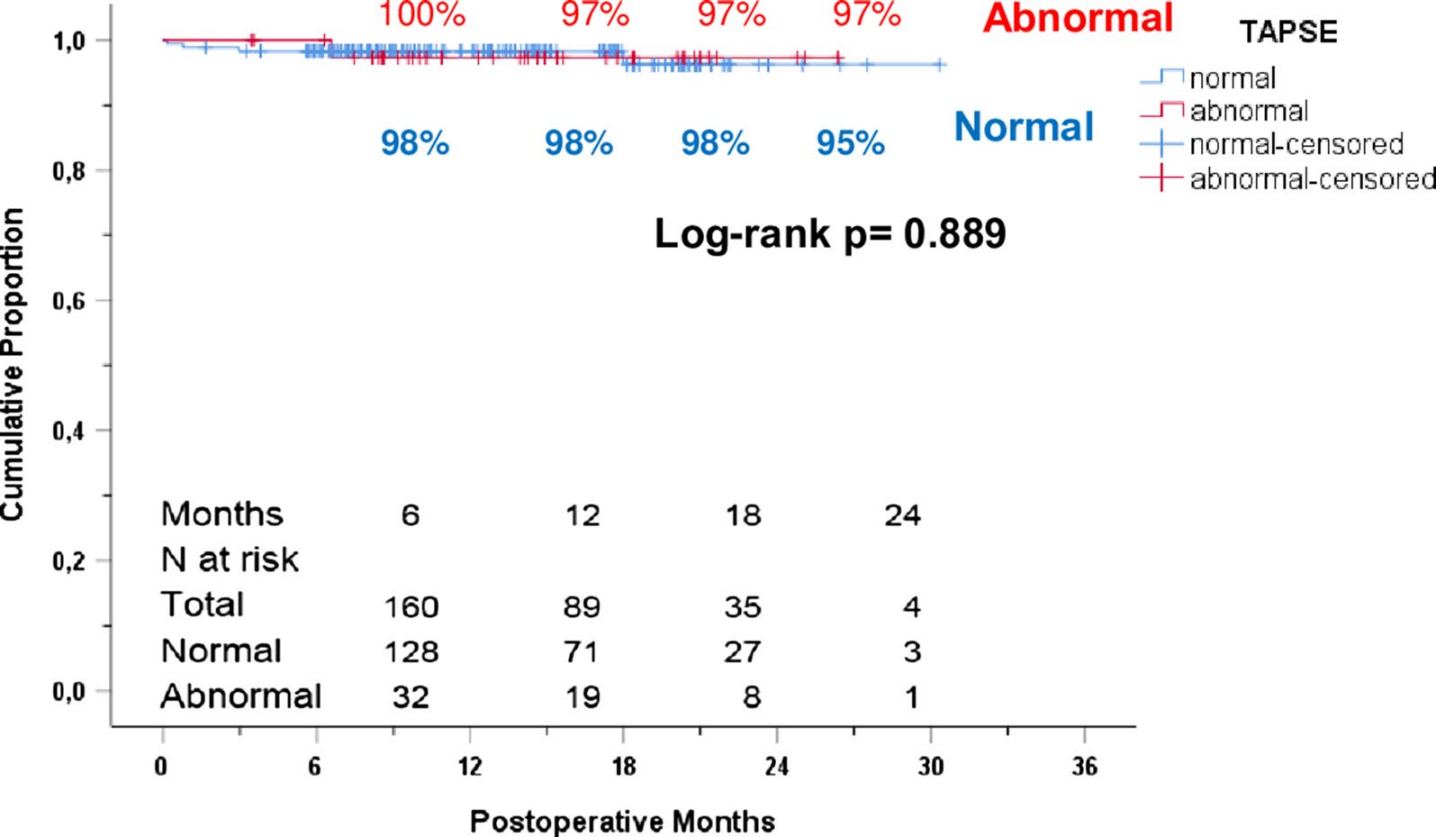
.

Predictors of known reduced right heart function, such as higher prevalence of significant preoperative AF (OR, 5.68; 95% CI, 2.79–11.58), advanced age (OR, 1.04; 95% CI, 1.00–1.08), higher EuroScore II (OR, 1.29; 95% CI, 1.01–1.63) appeared to be predictors for POAF. Moreover, larger LA diameter (odds ratio, 1.11; 95% CI, 1.01–1.21), abnormal TAPSE (OR, 2.61; 95% CI, 1.30 to 5.24), higher level of ADMA (odds ratio, 19.86; 95% CI, 1.71–230.76) and more arterial and venous grafts, respectively (OR, 1.77; 95% CI, 1.02–3.03 and OR, 1.83; 95% CI, 1.10–3.03) appeared to be significant risk for POAF (≤ 30 d). Abnormal TAPSE (OR, − 2.00; 95% CI, − 0.43 to 0.10) and larger RA (OR 2.05, 95%CI 0.01–0.46) appear to influence the odds of prolonged LOS (Fig. 1).

## Discussion

Our present study found that patients with reduced preoperative right ventricular function (TAPSE < 20 mm) showed significant impairment of the contractile apparatus utilizing our experimental model of skinned human right auricular fibers. A significant reduction in calcium-induced force values was observed at every force step generated by increasing calcium concentration. For TAPSE, cut-off point of < 16 mm according to guidelines, predicted impaired RV function with high specificity but low sensitivity [7]. Therefore, we examined patients with a TAPSE of 16–20 mm to ensure the presence of impaired RV function, which in turn, may also reflect RV myofilament involvement.

Our data show that experimental force development significantly decreases in patients with moderate to severe RV dysfunction. These results confirm that even in the absence of clinical symptoms, right heart dysfunction appears to be present in elective CABG patients. Thus, this subpopulation of “asymptomatic” surgical candidates may have a priori a greater incidence of developing RV failure. Available evidence does suggest that chronic ischemia of the inferior, lateral LV walls and ventricular septum increase the likelihood of RV dysfunction [14–19]. This might aggravate immediately after CABG, due to the fact that the ventricular septum, which is important for RV function is prone to inadequate myocardial protection in 40–80% of cardiac surgery patients [14–19].

Another finding is that DM, preoperative Afib, higher EuroScore II, lower albumin level, decreased GFR, and reduced LVEF were associated with reduced right heart function, which is in concert with literature (20–24). Our previous study demonstrated that there is a significant force decrease in right auricular myofilaments in patients with type 2 diabetes compared without [9]. Similarly, Patschneider et al. showed that DM as well as prediabetes is associated with decreased RV endsystolic and enddiastolic volumes as well as stroke volumes, indicative of early-onset RV impairment prior to symptom onset [20, 21]. Post-translational modification (O-linked ß-N-acetylglucosamine modification) in DM patients may have contributed to changes in calcium sensitivity, which is a measure of impaired contractile force development [9].

Gorter and coworkers demonstrated that Afib is associated with right heart dysfunction as result of decreased RA emptying fraction and reduced RA reservoir strain [22]. Similarly, we had earlier demonstrated significant reduced calcium induced force values after CABG were associated with new-onset POAF [10].

Recent studies have described the association of impaired renal function and decreased serum albumin levels in right-sided HF, which may be the result of subclinical remodeling process of the right atrium and ventricle [23, 24]. In sum, right ventricular dysfunction is a multifactorial disease with clustered and interactive risk factors, which could be detected at the level of the myofilament apparatus by means of a reduced calcium induced force value capacity.

To date, right-sided HF pathophysiology in symptomatic CHD for CABG remains unknown and there is no ability to determine who is at risk of progression or who will recover after CABG. To overcome these pitfalls, we analyzed a series of candidate markers for fibrosis and inflammation in baseline serum blood samples and correlated these factors with reduced right heart function. Significantly higher normalized concentrations of Pentraxin-3 in patients with reduced TAPSE vs. with normal TAPSE was significantly positively associated with reduced right heart function. Pentraxin-3 is a protein mediator of innate immunity produced by VSMCs and ECs [25] and observed predominantly in patients with impaired diastolic function, metabolic syndrome, myocarditis and chronic HF [26, 27]. Regarding indices of RV function, Pentraxin-3 is reported to modify RV adaption with resultant greater RV mass and larger RV end-diastolic volume independent of LV morphologic changes, suggesting a RV-specific relationship [27]. We also found ADMA significantly increased in patients with reduced TAPSE. ADMA is an analogue of L-arginine and a naturally occurring product of metabolism found in human circulation [28]. Elevated levels of ADMA inhibit NO synthesis, in turn, impair endothelial function and thus promote atherosclerosis. ADMA levels are increased in people with hypercholesterolemia, atherosclerosis, hypertension, chronic heart failure, diabetes mellitus and chronic renal failure [28]. A number of studies have reported ADMA as a novel risk marker of cardiovascular disease [28]. Increased levels of ADMA have been shown to be the strongest risk predictor, beyond traditional risk factors, of cardiovascular events and all-cause and cardiovascular mortality in CAD [28]. Accumulation of ADMA in chronic systolic heart failure was observed to be associated with presence of LV diastolic dysfunction as well as unfavorable pulmonary hemodynamics in patients with idiopathic pulmonary artery hypertension, however a direct association with decreased right heart function without these comorbidities was not shown thus so far [28, 29]. Our findings have revealed that increased ADMA levels, detected in patients with reduced right heart function might be associated with reduced renal function or prevalent DM (traditional risk factors for atherosclerosis). ADMA, in turn, may regulate NO expression in the pathogenesis of atherosclerosis [28]. Of importance in symptomatic CAD patients prior to CABG is the relationship of ADMA to RV dysfunction. This possible link remains to be elucidated independent of comorbidities.

In this study, reduced right heart function had no impact on clinical outcome parameters aside from significant longer hospital stay, which is inevitable in patients with reduced right heart function [2]. Reduced right heart function did not influence survival in our patient cohort; this might be due to either short follow up of 14 months or the fact that the majority of patients in the reduced TAPSE group belonged to the “grey zone “of right heart dysfunction (TAPSE 17–20 mm).

## Limitations

Several limitations must be noted. First, the sample size is still too small to draw conclusions concerning predictors for postoperative right heart impairment and follow-up in patients with CAD undergoing CABG procedure. Second, the higher incidence of diabetes and Afib in the group with reduced TAPSE might have also influenced the contractile capacity of the skinned fibers. Third, operator-specific treatment of the tissue samples with possible damage to the trabeculae cannot be excluded, although harvesting protocols were designed with a high degree of standardization. Furthermore in the scientific statement from the American Heart Association on evaluation and management of right-sided heart failure [30] cardiac MRI has become the gold standard for quantitative noninvasive measurement of RV dimensions and EF since cardiac MRI offers 3-dimensional imaging of the entire heart,

However, TAPSE has been established as an easy and fast obtainable screening method for right heart function with good correlation with more sophisticated techniques estimating RV systolic function (radionuclide-derived RV EF or 2D RF EF) [6]. Our patients were identified from a prospective, IRB-approved database, as such, might be subject to selection bias. By design, our study focused on elective on pump CABG without valve pathologies, reoperations, emergency indications or pulmonary hypertension of any origin. This design provided some homogeneity in order to evaluate similar clinical characteristics when comparing other published studies. Despite the nature of our study, our analysis may serve as catalyst for future mechanistic and observational studies to define predictors for impaired right heart function in patients with CAD.

## Conclusions

These preliminary data showed that a TAPSE of < 20 mm is associated with signs of right heart dysfunction at the level of myofilament apparatus. Increased Pentraxin-3 levels were associated with a TAPSE < 20 mm assuming that myofilament function is already decreased in a low normal range of TAPSE, which is echocardiographically supposed to be “normal” and could help identify patients at risk for right dysfunction immediately after CABG. However, larger studies and longer follow-up are mandatory to confirm our findings and delineate their clinical impact.

## Data Availability

The datasets used and analyzed during the current study are available from the corresponding author on reasonable request.

## Abbreviations

Afib: Atrial fibrillation
BMI: Body mass index
CABG: Coronary artery bypass grafting
DM: Diabetes mellitus
IL-6: Interleukin-6
LA: Left atrium
LAA: Left atrial appendage
LOS: Length of stay
LVEF: Left ventricular ejection fraction
N Acyl-SDMA: Symmetric dimethylarginine
NT-ProBNP: N-terminal pro brain natriuretric peptid
pCa: Logarithmic calcium concentration
RA: Right atrium
RAA: Right atrial appendage
SR: Sinus rhythm
TAPSE: Tricuspid annular plane systolic excursion
TGF ß: Transforming Growth Factor ß
